# A Detector for Premature Atrial and Ventricular Complexes

**DOI:** 10.3389/fphys.2021.678558

**Published:** 2021-06-16

**Authors:** Guadalupe García-Isla, Luca Mainardi, Valentina D. A. Corino

**Affiliations:** Biosignals, Bioimaging and Bioinformatics Laboratory (B3Lab), Department of Electronics, Information and Bioengineering (DEIB), Politecnico di Milano, Milano, Italy

**Keywords:** machine learning, ECG diagnosis, atrial fibrillation, beat classifier, supraventricular ectopic beat, premature ventricular contractions, premature atrial contractions, stroke

## Abstract

The relationship between premature atrial complexes (PACs) and atrial fibrillation (AF), stroke and myocardium degradation is unclear. Current PAC detectors are beat classifiers that attain low sensitivity on PAC detection. The lack of a proper PAC detector hinders the study of the implications of this event and its monitoring. In this work a PAC and ventricular detector is presented. Two PhysioNet open-source databases were used: the long-term ST database (LTSTDB) and the supraventricular arrhythmia database (SVDB). A combination of heart rate variability (HRV) and morphological features were used to classify beats. Morphological features were extracted from the ECG as well as on the 4th scale of the discrete wavelet transform (DWT). After feature selection, a random forest algorithm was trained for a binary classification of PAC (*S*) vs. others and for a multi-labels classification to discriminate between normal (*N*), *S* and ventricular (*V*) beats. The algorithm was tested in a 10-fold cross-validation following a patient-wise train-test division (i.e., no beats belonging to the same patient were included both in the test and train set). The resultant median sensitivity, specificity and positive predictive value (PPV) were 99.29, 99.54, and 100% for (*N*), 95.83, 99.39, and 35.68% for (*S*), 100, 99.90, and 79.63% for (*V*). The proposed method attains a greater PAC and ventricular beat sensitivity and PPV than the state-of-the-art classifiers.

## 1. Introduction

Premature atrial complexes (PACs) have always been considered benign. However, several recent studies link them to high risk of developing atrial fibrillation (AF) and stroke (Binici et al., 2010; Gladstone et al., 2015; Huang et al., 2017).

About 25–30% of ischemic strokes remain unexplained (cryptogenic) (Gladstone et al., 2015). One of the possible causes is that the thromboembolic events are caused by occult or silent AF. AF is the most common prevalent arrhythmia, affecting around 2% of global population. When AF is present without any perceived symptoms that enable its diagnosis, it is denominated silent AF. Prediction of the appearance of these episodes of AF could reduce the incidence rate of cryptogenic strokes. Several recent studies link frequent PACs to first time appearance of AF (Thong et al., 2004; Binici et al., 2010; Chong et al., 2011; Suzuki et al., 2013). Others have studied PACs as the possible direct reason for stroke (Huang et al., 2017). Furthermore, frequent PACs have been studied as a measure of cardiac tissue deterioration (Binici et al., 2010; Chong et al., 2011; Larsen et al., 2015; Huang et al., 2017) and as a possible cause for left ventricular remodeling (Pacchia et al., 2012).

All these studies point at the important and undervalued impact PACs may have on the cardiac electrophysiogy. However, manual beat annotation of long-term electrocardiogram (ECG) recordings is extremely time consuming and requires of specialized professionals. A PAC detector with high sensitivity able to assume this task is still missing. Such a detector would enable to study PAC implications in AF onset and cardiac tissue remodeling. It could be used to monitor patients for the occurrence of frequent PACs and determine stroke risk or possible silent AF or short paroxysmal AF (PAF) episodes. In addition, it could enhance the performance of arrhythmia detectors as PAC beats tend to increase AF false positives (Langley et al., 2012; Sörnmo et al., 2018).

To the extent of our knowledge, no proper detector explicitly designed for PAC is present in literature, most PAC detectors are actually beat classifiers (Llamedo and Martinez, 2012; Luz et al., 2016) that attain low PAC detection sensitivity. In this work we present a PAC detector not requiring any ECG delineation to extract morphological information. In addition, the extension of the methodology to also ventricular beat detection and beat classification is explored.

## 2. Materials and Methods

### 2.1. Data

Two PhysioNet public databases (Goldberger and Amaral, 2012) were used in this study: the long term ST database (LTSTDB) and supraventricular database (SVDB). Signals were 2-lead ECGs acquired at 250 and 128 Hz with a duration of 21–24 h and 30 min for the LTSTDB and SVDB, respectively. These databases were selected because they are the ones containing a higher number of PACs and manual beat annotations. The LTSTDB was originally built so as to represent a wide variety of ST segments. The SVDB contains a high number of supraventricular events. While the LTSTDB contains PACs together with different ST-segment variations, the SVDB contains a high number and variety of different possible PAC occurrences: bigeminy, trigeminy, and atrial runs. Both datasets were combined into a single dataset to use their complementary PAC representations for training and testing the model.

Table 1 gathers the number of beats per beat class in each database. In this study only 5 categories were originally considered as in Llamedo and Martinez (2011): Normal (*N*), Supraventricular (*S*), Ventricular (*V*), Junctional (*J*), and unclassifiable (*Q*) beats. We considered as PACs all *S* beats in which: atrial premature beats (*A*), aberrated atrial premature beats (*a*) and PACs (*S*) annotations were included. Instead, *V* comprehended the categories: premature ventricular contractions (*V*), fusion of ventricular and normal beats (*F*) and ventricular escape beats (*E*). Finally, *N* beats included normal beats *N*, bundle branch block beats (*B*) and atrial escape beats (*e*). *J* and *Q* classes were excluded for the successive analysis as they were underrepresented in both databases. Throughout the text, *N*, *S*, and *V* will be used to refer to the classifiers categories. As mentioned above, While *S* refer just to PACs, *V* represent ventricular beats (that include not only *V* but also *E* and *F*).

**Table 1:** PhysioNet and simplified beat annotations per database.

**Simp. Annotations**	**N**			**S**			**V**			**J**		**Q**
Beat Annotations	B	N	e	A	a	S	V	F	E	J	j	Q
LTSTDB	88,720	6,727,000	22	5,482	29	30,820	39,840	476	71	1	6	2
SVDB	1	162,100	0	0	1	12,090	9,930	23	0	9	0	80

*B stands for bundle branche block beat, N for normal beat, A for atrial premature beat, a for aberrated atrial premature beat, e for atrial escape beat, S for supraventricular premature or ectopic beat (atrial or nodal), V for premature ventricular contraction, F fusion of ventricular and normal beat, E for ventricular escape beat, J for nodal (junctional) premature beat, j for nodal (junctional) escape beat and Q for unclassifiable beat*.

### 2.2. Preprocessing

The preprocessing carried out was the same as in De Chazal et al. (2004). Firstly, all signals were resampled to 250 Hz to homogenize the sampling frequency of the datasets. Secondly, to obtain a baseline corrected signal, two median filters of 200 and 600 ms length were applied to obtain the baseline wander estimate which was then subtracted from the original raw one. Thirdly, a finite impulse response (FIR) low pass filter with cut off frequency of 35 Hz and equal ripple in the pass- and stop-bands was applied to remove powerline and high frequency noise. The full preprocessing was performed on MATLAB 2020a, The Mathworks Inc.

### 2.3. Feature Extraction

To classify each beat into one of the three categories considered in section 2.1, a set of features (185 in total) was extracted to describe two main properties of the ECG: the heart rate variability and wave morphology. To these features, the first and last 40 beats were not considered.

#### 2.3.1. HRV Features

For each individual beat, a set of features was computed taking into account the neighboring beats. *RR* intervals are defined as the distance of two consecutive *R* peaks of each beat. *dRR*s are instead the series of the difference of consecutive *RR*s, namely *dRR*_*n*_ = *RR*_*n*+1_ − *RR*_*n*_. Both the *RR* and *dRR* of the corresponding beat (*RR*_*i*_, *dRR*_*i*_), the previous beat (*RR*_*i*−1_, *dRR*_*i*−1_) and the following one (*RR*_*i*+1_, *dRR*_*i*+1_) were analyzed. Four different time windows were considered for the extraction of the heart rate variability (HRV) features: 1 or 5 min windows preceding the current beat; 2 or 10 min windows centered on the current beats. From each time window the mean and standard deviation of the RR intervals, along with the standard deviation of the dRR intervals, the percentage of successive interval differences greater than 10, 20, 30, 40, and 50 ms (pNN50) and the root mean square of successive differences (RMMSD) were computed. A total of 41 HRV features were measured.

#### 2.3.2. Morphological Features

Morphological information of the P wave, QRS complex, PR segments and the whole beat were extracted using, a fixed window. The window dimensions, using the *R* peak as reference (i.e., *t* = 0), for the ECG segments considered were: [–300, 40] ms for the P wave segment, [–70, 60] ms for the QRS complex, [–288, 0] ms for the PR interval and [–300, 250] ms for the whole beat (Censi et al., 2007). The following segments will be referred as the P wave, QRS complex and PR interval, respectively throughout the rest of the paper. However, it should be noted that as no ECG delineation is performed, the reported segments may not precisely account for these ECG regions (i.e., it is not an exact selection of the onset and offset of the ECG segment, but rather an approximate estimation). Nevertheless, the scope of this selection is to account for their intra-patient variability not to extract any precise parameter which could describe any of the ECG regions described above. Therefore, given that for the same patient the same ECG portion would be extracted for each of the mentioned segments, any variability produced by a premature atrial or ventricular beat, should be detected even if the ECG region is not accurately delineated. Prior extraction of the ECG segments, an intra-patient template was created using the neighboring beats. Three different templates were computed using 80 (40 prior and 40 posterior the beat of study), 20 (10 prior and 10 posterior the beat of study) and 4 beats (2 prior and two posterior the beat of study) each. Three templates were computed to represent the instant beat differences with respect to the short-term neighboring beats (4-beat template) and compare each beat with respect to the long-term (80-beat template) and the mid-term (20 beats). While the short-term could be especially useful for the detection of isolated PACs, the long and midterm could be more relevant in identifying PACs in bigeminy, trigeminy or in atrial runs.

The surrounding beats' segments were aligned through cross-correlation and then averaged. Outlier segments, according to the maximum cross-correlation value obtained for alignment, were excluded from the mean and thus, from the computation of the intra-patient template.

Once the intra-patient templates were computed, each beat of the subject was compared with the templates using cross-correlation. At the end of the process, for each beat, the following parameters were extracted and used as features:

- Maximum cross-correlation value of each segment with respect to the different intra-patient templates created with the neighboring beats (80, 20, and 4).
- Lag corresponding to the cross-correlation value described above.
- Median standard deviation of the beats used to create the intra-patient template.

The features enumerated above were computed for each lead of the ECG independently. A total of 72 morphological features were computed for each beat.

##### 2.3.2.1. Discrete Wavelet Transform

Morphological features were computed also on a filtered version of the ECG obtained through the discrete wavelet transform (WT). The WT for a continuous signal *s*(*t*) if defined as follows:

(1) $$\begin{array}{l} W_{s}s(b)=\frac{1}{\sqrt{s}}\int_{+\infty }^{-\infty }s(t)\psi (\frac{t-b}{s})ds,s>0 \end{array}$$

This transform maps the input signal into the time-frequency plane by means of the prototype wavelet function ψ(*t*), dependent of the scaling (*s*) and translation (*b*) parameters. Low values of *s* enable the WT to localize fast transitions, whereas higher values localize coarser changes instead. Instead, the translation parameter *b* correspond to their location (Martínez et al., 2004).

A computationally feasible version of the WT is the discrete WT (DWT) which discretizes the time-scale by means of a dyadic sampling i.e., *s* = 2^*k*^ and *b* = 2^*k*^*l* for *k, l* ∈ *Z*. The same implementation followed in Llamedo and Martinez (2011) and implemented in Demski and Soria (2016) was performed. In Llamedo and Martinez (2011) *b* = *l* for *l* ∈ *Z* so as to maintain the same sampling frequency in all scales. A quadratic spline was used as prototype wavelet ψ(*t*), retaining ECG information at determined scales (Martínez et al., 2004). The fourth scale of the DWT [*W*_4_*s*(*l*)] retains useful information of the ECG (Llamedo and Martinez, 2011). This ECG decomposition was also used to extract morphological information described above in the *Morphological features* section using the mentioned implementation characteristics. The resultant set of morphological features were composed by the same intra-patient cross-correlation information but computed using the filtered ECG signal and the [*W*_4_*s*(*l*)] ECG decomposition. A total of 72 DWT morphological features were computed for each beat.

### 2.4. Model Definition and Training

The selected classification model was a random forest (RF) evaluated in a patient-wise, 10-fold cross-validation i.e., no beats belonging to the same patient were included in the training and test set.

#### 2.4.1. Train-Test Dataset Definition

The dataset was divided into 10 different subsets. Given the unbalance occurrence of *S* among different patients, the data subsets were conformed so as to maintain a similar proportion of *S* in each Kfold. At each iteration of the cross-validation, 9 of the 10 subsets were used for training and the remaining was used to test the performances. To prevent patient bias during training, a 10,000 upper bound limit was set for the number of beats of each class used for training the algorithm.

#### 2.4.2. Feature Processing

To remove possible outliers an upper and lower bound was set for the features containing *RR* and *dRR* information. Values out of the established boundaries were reevaluated as the lower or upper limit (depending on which threshold they exceeded). Based on the cardiac refractory period, the minimum *RR*_*min*_ considered was 250 ms. On the other hand, if we accept 35 beats per minute (bpm) as the lowest possible heart rate (considering an extreme case of bradycardia), the corresponding *RR* interval would be 60*s*/35*bmp* = 1, 714.3*ms*. However, it is known that after a PAC a refractory pause is caused due to the depolarization of the sinoatrial node, and that the maximum this pause can be is double the normal *RR*. Therefore, the maximum *RR* considered was *RR*_*max*_ = 3428.6*ms* (Sörnmo and Laguna, 2005). The dependence among the different variables was computed using Pearson's correlation coefficient. Correlation sequence were normalized so that the autocorrelations at zero lag were equal to one. In addition, features with a variance lower than 0.05 were excluded. Finally, a z-score transformation was applied to the remaining features.

#### 2.4.3. Model Definition

RF is a supervised tree-based ensemble machine learning model trained with the "bagging" method. The concept behind bagging is that the combination of several weak simple classifiers can lead to high performance. RF builds a strong classifier by adding together simple decision trees. A strong advantage of this methodology is its resistance toward over-fitting which is of great importance to reduce patient and database-dependent bias and ensuring the extrapolation of the model to other scenarios. A first train-test patient-wise split was performed for hyper-parameter tuning. The train set was composed by a random group of patients summing up to the 80% of *S* from the whole dataset, while the test set were all the remaining ones. A first random hyper-parameter search was carried out to prove the most suitable ranges. Grid hyper-parameter search was performed based on the results of the first random search. The best-performing hyper-parameters chosen were: number of estimators = 500, minimum samples for a split = 10, minimum samples for a leaf =2, maximum tree depth =20 and sample replacement in bootstrap aggregation = *False*. RF was implemented in Python 3.8 version using the Scikit-learn library version 0.24.0.

**Figure 1:**
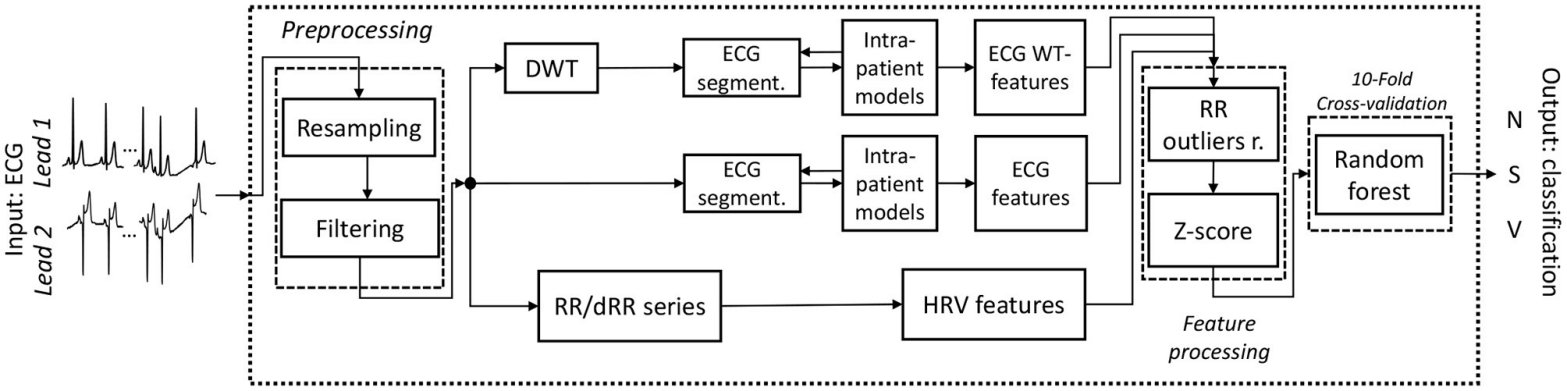
Outline of the final working classifier pipeline. The input consists of a 2-Lead ECG and the output on the classification of the beat of one of the three categories *N, S* and *V. Outliersr*. stands for outliers removal.

An outline of the full final model pipeline described is shown in Figure 1.

The model was both assessed as a PAC detector or binary classifier to discriminate *S* vs. *Other*, and as a multi-class classifier for *N*, *S* and *V* discrimination following the same beat classifiers strategy present in literature. As stated above, *S* category included PAC with different notations across the two databases used (*A*, *a* and *S*), while *Other* included both *N* (formed by *B*, *N* and *e*) and *V* (formed by *V*, *F* and *E*) categories.

## 3. Results

### 3.1. Total Features Computed

**Figure 2:**
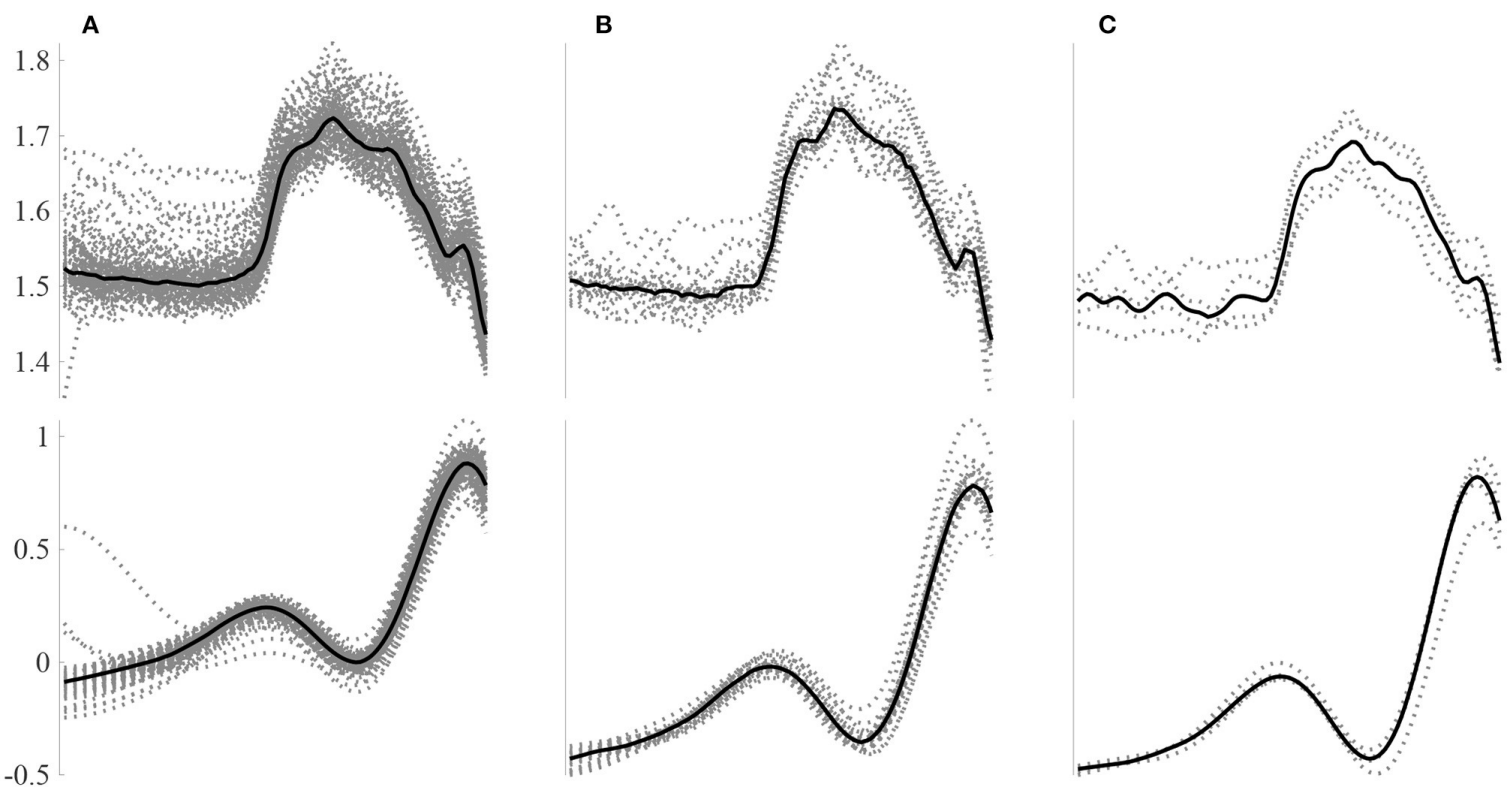
Example P-wave intra-patient models built using a different number of surrounding beats for the *raw* signal (top) and the *W*_4_*s*(*l*) DWT decomposition. Intra-patient models built using **(A)** 80, **(B)** 20, and **(C)** 4 beats.

A total of 185 features were computed for 6126250 *N*, 48,032, *S* and 40,312 *V* beats: 41 HRV features plus 144 morphological features (from which 72 of the temporal signal and 72 of the DWT). For each beat, a total of 48 intra-patient models were computed: 4 segments (whole beat, P-wave, PR segment, QRS complex), 3 beat windows used to construct each model (80, 20 and 4 beats) and 2 leads for both the raw signal and the [*W*_4_*s*(*l*)] of the DWT. Figure 2 presents an example of P-wave intra-patient models computed with 4, 20, and 80 neighboring beats for the original and the WT of the ECG.

**Figure 3:**
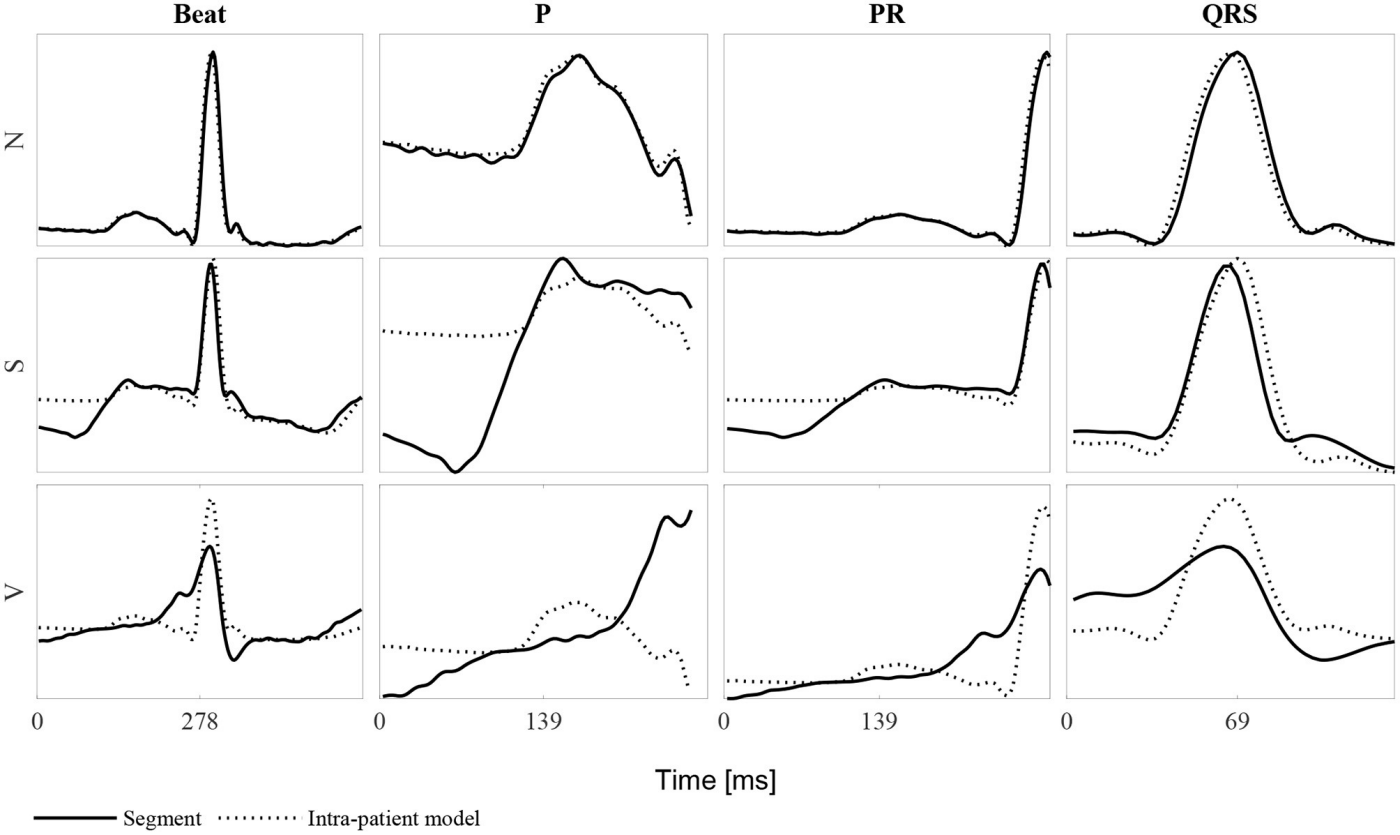
Example of each of the beat classes *N*, *S*, and *V* for the whole beat, the P wave, PR segment and QRS complex. Each row represent one of the three beat types and each column the mentioned ECG segments. Continuous lines represent the beat segment itself whereas dashed lines represent the corresponding intra-patient models built with the 40 beats before and after the beat of study (80-beat intra-patient model).

Figure 3 shows an example of an *N*, *S*, and *V* beat and the corresponding P wave, PR interval and QRS complex. The corresponding intra-patient model built using the 40 anterior and posterior beats is also shown. It can be noted that for the *N* beat, the P wave, PR segment and QRS complex match almost perfectly the intra-patient model. In contrast, the *S* beat's P wave differs considerably from the intra-patient model, the PR segment slightly differs and the QRS complex almost matches it. Finally, all *V* intervals differ from the corresponding intra-patient models. Ventricular beats are not usually accompanied by a prior P wave. This is the reason why it does not match the neighboring P waves.

### 3.2. Most Relevant Features

Feature importance was analyze at each Kfold for the binary and multi-label classification. The top 10 most important features according to the random forest model for each Kfold of the cross-validation are gathered in Table 2 for the bi-label and multi-label approach. A total of 20 features conformed the 100 most important features (10 for each Kfold) for the multi-label classification and 18 for the binary one.

**Table 2:** The ten most relevant features for each cross-validation KFold for the bi-label and the multi-label classification.

**Bi-label classification**	**Multi-label classification**
*dRR*_*i*_	*dRR*_*i*_
Beat cross-corr. DWT, L2, 20-beat intr.temp	Beat cross-corr. DWT, L2, 20-beat intr.temp
Beat cross-corr. L1, 20-beat intr.temp	Beat cross-corr. L1, 20-beat intr.temp
	Beat cross-corr. L1, 4-beat intr.temp
Beat cross-corr. L1, 80-beat intr.temp.	Beat cross-corr. L1, 80-beat intr.temp.
Beat cross-corr. L2, 4-beat intr.temp	Beat cross-corr. L2, 4-beat intr.temp
Beat cross-corr. L2, 80-beat intr.temp.	Beat cross-corr. L2, 80-beat intr.temp.
*dRR*_*i*+1_	*dRR*_*i*+1_
*dRR*_*i*−1_	*dRR*_*i*−1_
P cross-corr. L1, 80-beat intr.temp.	P cross-corr. L1, 80-beat intr.temp
P cross-corr. L2, 80-beat intr.temp.	P cross-corr. L2, 80-beat intr.temp.
	PR cross-corr. L1, 80-beat intr.temp
QRS cross-corr. DWT, L2, 20-beat intr.temp.	QRS cross-corr. DWT, L2, 20-beat intr.temp.
QRS cross-corr. DWT, L2, 4-beat intr.temp.	QRS cross-corr. DWT, L2, 4-beat intr.temp.
QRS cross-corr. L1, 4-beat intr.temp.	QRS cross-corr. L1, 4-beat intr.temp.
QRS cross-corr. L1, 80-beat intr.temp.	QRS cross-corr. L1, 80-beat intr.temp.
QRS cross-corr. L2, 20-beat intr.temp.	QRS cross-corr. L2, 20-beat intr.temp.
QRS cross-corr. L2, 4-beat intr.temp.	QRS cross-corr. L2, 4-beat intr.temp.
QRS cross-corr. L2, 80-beat intr.temp.	QRS cross-corr. L2, 80-beat intr.temp.
*RR*_*i*_	*RR*_*i*_

*Intr.temp stands for intra-patient template. L1 and L2 refer to leads 1 and 2, respectively*.

The most important features were shared between both bi- and multi-label approaches with the exception of Beat cross-correlation of Lead 1 (L1) with 4-beat intra-patient template (intr.temp.) and PR cross-correlation of L1 with 80-beat intr.temp. that were only included as the 10 most important for the multi-label classifier and not for the binary one. The most relevant features were those accounting for *RR* disturbances, QRS complex and Beat morphology for the temporal ECG signal. Only three DWT morphological features were included as top features.

### 3.3. Classifier Performance

The proposed model was evaluated for a binary classification (*S* vs. *Other*) for evaluating explicit PAC detection and for a multi-class classification, evaluating the proposed model as a beat classifier. It should be noted that, the number of *S* per patient varied considerably among patients and thus, not all had the same weight when accounting the classifiers' performance. Therefore, the performance of the model could be assessed in two ways: taking into consideration each beat as a separate sample, regardless of the patient (Table 3), or by averaging the accuracy, sensitivity and specificity values of every single patient regardless of their number of *N*, *S*, and *V* beats (Tables 4–6). In addition, patient-wise performance median and percentile values were provided considering a patient division by database to enhance comparability with other studies and to provide information about the database dependencies on the results reported.

**Table 3:** Classifier performance considering single beats regardless of the patient.

	**#Beats**	**Acc. (%)**	**Se. (%)**	**Sp. (%)**	**PPV (%)**
*S* (binary class.)	48,032	98.15	89.83	98.78	35.23
Normal (N)	6,126,250	97.88	97.90	96.57	99.85
Supraventricular (S)	48,032	98.30	92.65	98.34	30.30
Ventricular (V)	40,312	99.51	95.69	99.53	57.87

*Acc., Se., Sp. stand for accuracy, sensitivity, specificity, respectively*.

**Table 4:** Patient-based classifier performance, median (IQR range) for the binary classification (*S*–*Other*).

	**LTSTDB (S)**	**SVDB (S)**	**LTSTDB+SVDB (S)**
#Pat.	63	72	135
Accuracy (%)	99.84 (99.53–99.95)	97.82 (92.34–99.49)	99.48 (96.31–99.87)
Sensitivity (%)	94.12 (85.85–99.98)	91.78 (75.68–98.05)	92.86 (82.60–99.63)
Specificity (%)	99.85 (99.53–99.95)	98.63 (94.85–99.66)	99.57 (98.13–99.91)
PPV (%)	21.21 (3.36–45.07)	66.67 (31.19–82.88)	40.87 (14.90–73.36)
NPV (%)	99.99 (99.99–100)	99.77 (99.26–99.97)	99.99(99.70–100)

*Values are expressed as median (25–75th) percentile*.

**Table 5:** Patient-based classifier performance, median (IQR range) for the multi-class classification.

	**Normal (N)**	**Supraventricular (S)**	**Ventricular (V)**
#Pat.	139	132	123
Acc. (%)	99.05 (95.40–99.74)	99.35 (95.78–99.84)	99.87 (99.34–99.99)
Se. (%)	99.29 (94.96–99.78)	95.83 (87.50 -100)	100 (95.84–100)
Sp. (%)	99.54 (96.62–100)	99.39 (95.84–99.87)	99.90 (99.53–100)
PPV (%)	100 (99.78–100)	35.68 (9.63–69.57)	79.63 (15.71–97.39)
NPV (%)	62.61 (27.61–84.73)	99.99 (99.73–100)	100 (99.96–100)

*Values are expressed as median (25–75th) percentile. Acc., Se., Sp. stand for accuracy, sensitivity, specificity, and respectively*.

**Table 6:** Patient-based classifier performance, median (IQR range) for the multi-class classification.

	**LTSTDB**		**SVDB**	
	**S**	**V**	**S**	**V**
#Pat.	63	60	72	66
Acc. (%)	99.76 (99.37–99.92)	99.93 (99.68–99.99)	97.13 (90.49–99.39)	99.76 (98.98–99.95)
Se. (%)	95.65 (88.89 -100)	99.81 (96.83 -100)	96.20 (84.70–100)	100 (85.74–100)
Sp. (%)	99.76 (99.37–99.93)	99.95 (99.76–99.99)	97.74 (91.46–99.54)	99.84 (99.44–100)
PPV (%)	13.36 (2.29–44.56)	45.83 (2.72–91.73)	57.58 (25.03–80.73)	93.66 (67.95–98.87)
NPV (%)	99.99 (99.99–100)	100 (99.99–100)	99.84 (99.43–100)	100 (99.90–100)

*Values are expressed as median (25–75th) percentile. Acc., Se., Sp. stand for accuracy, sensitivity, specificity, and respectively*.

#### 3.3.1. Binary Classification

The first row of Table 3 represent the accuracy, sensitivity, specificity, positive predictive value (PPV, and negative predictive (NPV) value results for *S* detection from a beat-wise perspective; considering each beat as a sample independently of the patient it came from. Although sensitivity values were slightly lower than those reported in the same table for multi-label classification, the PPV was higher. Table 4 instead present results from a patient-wise performance. Following the interquartile range (IQR) of the PPV presented in Table 4 a high patient-dependent influence can be intuited. Low PPV values even with high sensitivity and specificity are given by the extreme class imbalance of the dataset.

#### 3.3.2. Multi-Label Classification

**Figure 4:**
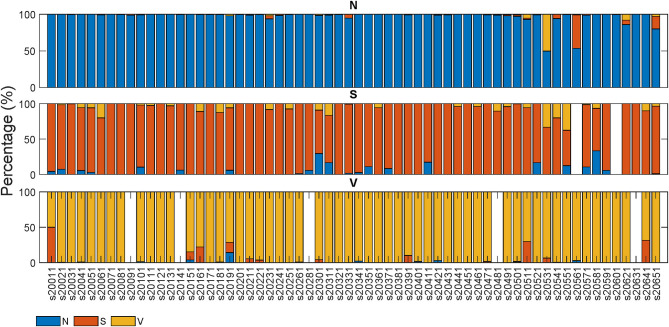
Classification percentage of each of the LTSTDB signals' beats. Each sub-graph represents the classification distribution of the beats of the three classes considered: N, S, and V. Each bar in each sub-graph represents the total number of beats of that class of a single patient and how they have been classified (in percentage). The *x*-axis represent the different patient IDs.

**Figure 5:**
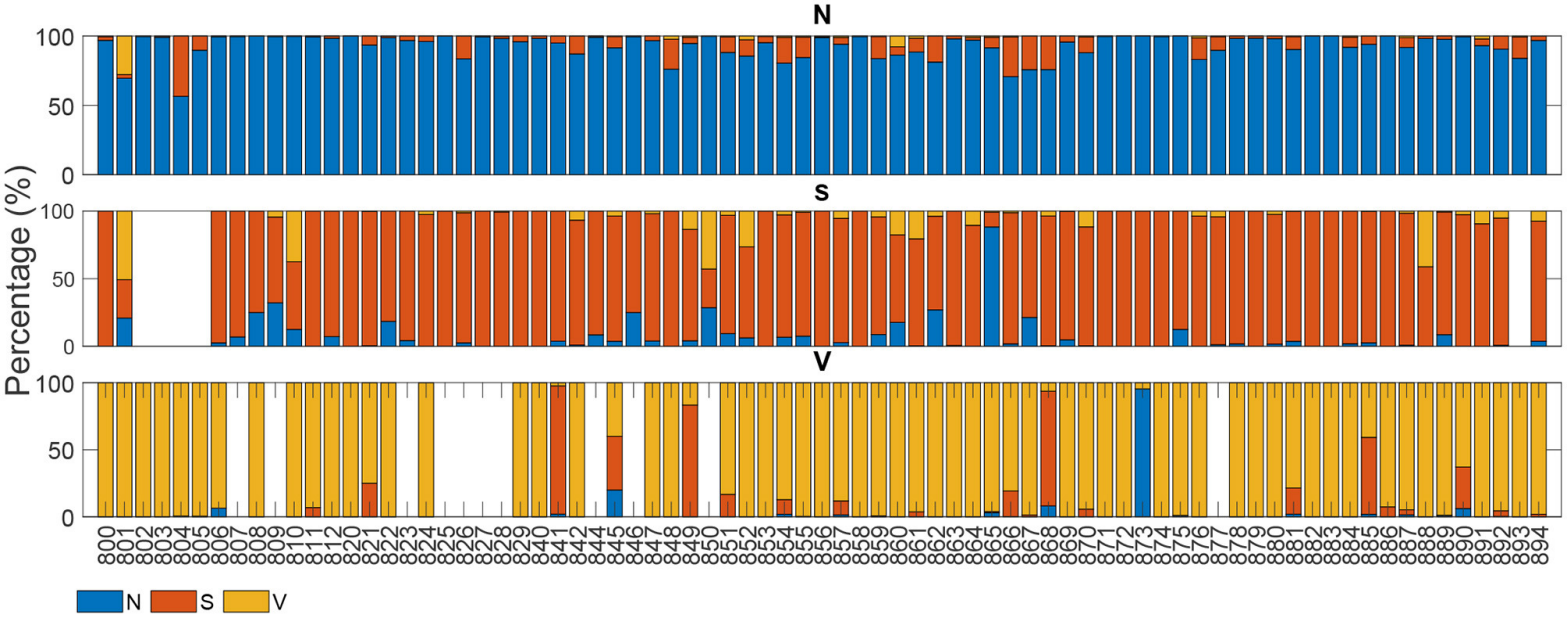
Classification of each of the SVDB signals' beats. Each sub-graph represents the classification distribution of the beats of the three classes considered: *N*, *S*, and *V*. Each bar in each sub-graph represents the total number of beats of that class of a single patient and how they have been classified (in percentage). The *x*-axis represent the different patient IDs.

**Figure 6:**
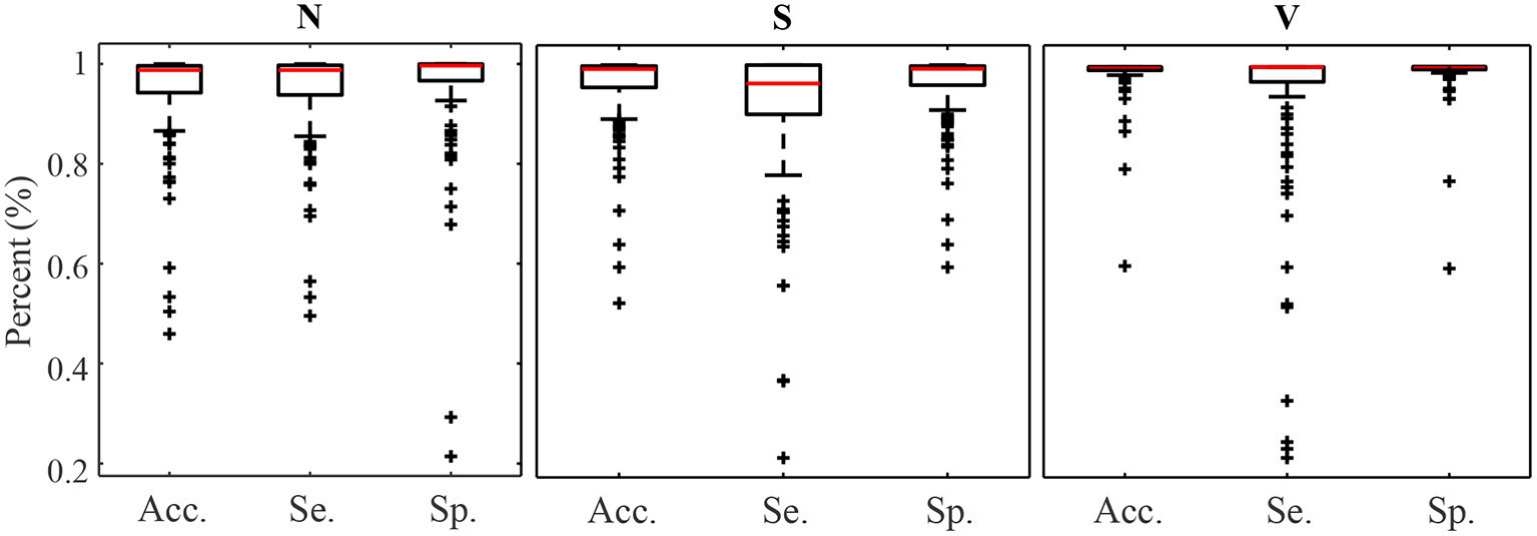
Accuracy Sensitivity and Specificity box plot for *N*, *S*, and *V* detection calculated for each patient independently.

Figures 4, 5 display the classification distribution for the LTSTDB and the SVDB, respectively. Each of the three sub-graphs shows the classification of one of the three beat categories. Bars represent the classification distribution of individual patients for that specific beat type, in percentage. For example from patient *s*20301, Figure 4 shows that all *N* beats (top subgraph) were correctly classified, around 20 and 10% of *S* beats (middle sub-graph) were misclassified as *N* and *V*, respectively and <10% of all *V* beats (bottom sub-graph) were misclassified as *S*. From both figures, it can be noted that most of the beats were correctly classified in all patients as it can be also appreciated by the overall results reported in Table 5. From results in Figures 4, 5 it can be derived that *S* and *V* misclassifications have a strong patient-dependent component. Figure 6 shows the classification distribution computed for each patient independently as in Table 5. The presence of outliers show that even if the classifier attained very high performance for most patients, for some of them it failed to properly classify into the three categories. *S* sensitivity attained the highest inter-patient variability values.

Results in Table 5 show the median and IQR of the accuracy, sensitivity and specificity values for the patient-wise *N*, *S*, and *V* classification performance. The three classes attained a sensitivity and specificity higher than 99%, with the exception of *S* sensitivity that was 95.83%. *S* sensitivity also attained a higher IQR than the rest of the categories. PPVs for the *S* class were inferior to those of the other categories, influenced by the presence of false positives and class imbalance. Multi-class *S* sensitivity was slightly higher than that of the binary classification. PPVs instead were superior and with a lower IQR for the binary classification than for the multi label one.

Finally, Table 7 shows the confusion matrix. It can be observed that the majority of false negatives for *N* and *V* were *S* whereas for *S* most of the false negatives were *N*.

**Table 7:** Confusion Matrix of total classified beats in percentage.

	**Normal (N)**	**Supraventricular (S)**	**Ventricular (V)**
Normal (N)	97.52%	1.94 %	0.54%
Supraventricular (S)	4.89%	93.44%	1.67%
Ventricular (V)	0.69%	3.79%	95.52%

*The vertical and horizontal axis represent the true labels and predicted classes, respectively*.

## 4. Discussion

Explicit PAC detection in the ECG has not gained great attention, as it can be noted by the low number of papers addressing solely this problem (Visinescu et al., 2004; Krasteva et al., 2006). Rather, extensive literature can be found regarding a broader beat classification into supraventricular, ventricular and normal categories (Llamedo and Martinez, 2012; Luz et al., 2016).

PACs in the ECG are characterized by two alterations: disruption of the *RR* sequence and distortion of the P wave morphology. While in Petrenas et al. (2017) four different types of PACs were described based on how they altered the *RR* interval, in Kistler et al. (2006) different P wave morphologies were explored depending on the PAC site of origin. The combination of HRV and morphological features included in the method proposed in this study aimed to take advantage of both characteristics.

Given that *R* peak detection is more robust against noise and less patient-dependent than morphological ECG information, *RR* interval-derived features are the most reliable to discriminate PAC from normal beats. However, ventricular beats generate *RR* sequence alterations similar to those induced by PACs and thus, they need to be distinguished from PACs based on the ECG morphology. The main appreciable differences on the ECG between PAC and ventricular beats lie on the QRS complex and P wave morphology. Nevertheless, whereas QRS distortions are of high amplitude and can typically be reliably distinguished, the P wave is more susceptible to noise and its morphology may be easily altered by external sources rather than by an electrophysiological disturbance.

The feature importance displayed in Table 2 is coherent with this, pointing at the main role *RR* intervals and QRS complex morphology features had on the *S* and *V* detection capacity. The consistency found between the most relevant features for the binary and the multi-class classifiers could be explained by the fact that the same ECG characteristics (*RR* intervals and QRS complex) can be used to distinguish both *S* and *V* classes from *N* as well as from themselves. Therefore, the features better representing these ECG characteristics were the ones attaining a higher relevance in both binary and multi-class classifiers.

Observing Table 2 one could intuitively guess that the model detects an *RR* alteration and it discriminates between *S* and *V* or *Other* (in the multi-label or binary approach, respectively) by checking if the QRS complex morphology is or not altered.

Even if the DWT has been proved useful for extracting relevant ECG information (Martínez et al., 2004; Llamedo and Martinez, 2012), according to Table 2 morphological features extracted from the DWT seemed to have a lower impact on the overall classifier's performance. While the morphological features obtained using the raw signal accounted for any morphological signal changes, morphological DWT features accounted for changes occurring only at a determined frequency band. Results suggest that the morphological changes induced in the 4th scale of the DWT captured by cross-correlation with respect to the intra-patient templates, were not as representative as those capturing morphological changes in the temporal signal.

It is known that ECG signals acquired from different patients have a considerable inter-patient variability. These dissimilarities hider the definition of universal measures that could serve as descriptors of eletrophysiological events (as PAC or *V*). As a result, ECG delineators and beat classifiers attaining high performance across different patients and databases are rather challenging. By extracting morphological features that do not depend on precise measures but on the analysis of the evolution of the ECG signal itself, the inter-patient dissimilarity problematic is bypassed. In contrast, classifiers as De Chazal et al. (2004) and Zhang et al. (2014) depended on a proper ECG delineation to extract morphological information.

Two approaches were taken to study the proposed model: a proper PAC detector by discriminating between two categories (*S*–*Other*) and a beat classifier to discriminate among three different classes (*N*, *S*, and *V*). Sensitivity values increased slightly for multi-label approach but with a reduction in PPV in comparison to the binary classification. Although PAC detection was the main target of the development of this model, results obtained for the multi-label approach shows that the classifier can be successfully adapted to the detection of also ventricular beats without major performance degradation in PAC detection performance.

Great care was taken in this work so as to not only maintain a balance among the three beat categories in the train set but also among the number of beats belonging to different patients, in order to avoid a patient-biased trained model. As it can be seen in the results in Figures 4, 5 as well as in Table 5, the detector performance varies among patients, evidencing the strong inter-patient influence on discriminating different beat types. One factor contributing to this could be the differences between lead placement on patients for acquiring Holter recordings. Different lead placement for Holter monitoring would influence amplitudes for the ECG segments, specially for regions as the P-wave. In patients with ECG signals attaining a lower P-wave amplitude, morphological distortions would be less evident and thus more difficult to detect.

### 4.1. Related Work

From the published methods, an initial distinction can be made based on if a proper patient-wise train-test division was made. As demonstrated by Llamedo and Martinez (2012), there exists a strong bias introduced in algorithms trained and tested with beats belonging to the same patients. A second distinction can be made based on the database used for testing the methodology. The Association for the Advancement of Medical Instrumentation (AAMI) guidelines recommend the open-source MITBIH Arrhythmia database available at Physionet as a common framework for reporting performance as it is the only one that contains the five superclasses of arrhythmias. However, as discussed by Luz et al. (2016) this database is highly unbalanced and provides misleading results about supraventricular and ventricular beats detection. A standardized train-test division of the MITBIH arrhythmia database was proposed by De Chazal et al. (2004), which has been used by many authors as Yu and Chen (2007), Yu and Chou (2008), Mar et al. (2011), and Zhang et al. (2014). However, most *S* and *V* occur in single patients in both sets and extrapolation of the performance to other patients is rather doubtful. Llamedo and Martinez (2012) performed an exhaustive analysis about how the databases used for testing changed significantly the performance reported by the same methodology. Therefore, it is important to understand that comparison between algorithms is not trivial and that it should be interpreted with care.

De Chazal et al. (2004) used *RR* intervals and morphological information of the segmented ECG as features and linear discriminant (LDs) models as classifier. They used for training and testing the MITBIH arrhythmia database divided by the standard DB1 DB2 introduced by themselves. They reported a sensitivity of 75.9%, a PPV of 38.5% and a FPR of 4.7%. In two studies, Llamedo and Martinez (2011) and Llamedo and Martinez (2012) developed a classifier including *RR* interval and morphological features from different scales of the DWT. In a first study (Llamedo and Martinez, 2011) used a LD classifier (LDC) and tested their method on the DS2-Test set of the MITBIH obtaining a SVEB sensitivity of 77% and a PPV of 88%. In addition, they also tested the methodology on the whole MITBIH Arrhythmia database reporting a SVEB sensitivity of 61% and a PPV of 73%. In a second study (Llamedo and Martinez, 2012) they used up to 8 public databases, among which were the SVDB and the LTSTDB (containing the 82.01% of the total PACs) to train and test their model. They used their previously developed classifier together with and unsupervised clustering method to construct their model. In addition they enabled it to be assisted, semi-assisted or automatic. They obtained a sensitivity and a PPV of 76 and 43% in the full MITBIH in automatic mode that increased up to 89–88%, respectively, in assisted modality. Similarly in the SVDB they obtained 47 and 50% that increased to 74 and 79% sensitivity and PPV in automatic to assisted, respectively. Finally in the LTSTDB they obtained 50% and 8% sensitivity and PPV in automatic and 51 and 58% in assisted. To the best of our knowledge (Llamedo and Martinez, 2012) are the only ones reporting results using the SVDB and LTSTDB as test set.

The classifier presented in this work attained a sensitivity and PPV of 94.12 and 21.21% for the LTSTDB and 91.78 and 66.67% for the SVDB as shown in Table 4. Results for both databases were considerably higher in terms of sensitivity in comparison with those reported in Llamedo and Martinez (2012) for the automatic and assisted classification. PPVs were higher that the ones reported by Llamedo and Martinez (2012) only in the fully automatic mode. Nevertheless, PPV should be interpreted with care as, given the high class imbalance (of more than 2 orders of magnitude) *S* PPVs would increase if *S* sensitivity was equal to zero, thus not detecting PACs at all. Therefore, sensitivity values ought to be taken in consideration together with the PPV. The beat classifier presented by Llamedo and Martinez (2012) attains a higher PPV in the SVDB (76%) in the assisted mode. However, the reported sensitivity in the LTSTDB using that same methodology reaches only a 51% which would not make it suitable for PAC detection. It is evident that using the proposed methodology or the one presented by Llamedo and Martinez (2012) is a matter of trade-off with regard to the amount of false positives or false negatives as far as PAC detection is concerned.

On the other hand the sensitivity and PPV values obtained for *V* classification shown in Tables 5, 6 (100 and 93.66% for the SVDB and 99.81 and 45.83% for the LTSTDB) were superior to those reported by Llamedo and Martinez (2012) in the automatic mode (sensitivity and PPV of 82 and 54% percent for the SVDB and 43 and 11% for the LTSTDB). For the assisted mode they obtained a sensitivity and PPV of 88 and 90% for the SVDB and 95 and 99% for the LTSTDB. It should be noted that even if some performance values are higher for *S* or *V* detection for the assisted version of the classifier presented in Llamedo and Martinez (2012), this version requires of the intervention of a user to verify the final classification which could induced human errors as well as variability among users in the final classification. These results suggest that the proposed model could be used not only for PAC, but also for *V* detection, obtaining performance results for beat classification higher than those present in literature.

The proposed model enabled the detection of almost all PACs included in this study, implying an step ahead in PAC detection, as the available methods' sensitivity was always kept low (De Chazal et al., 2004; Yu and Chen, 2007; Yu and Chou, 2008; Llamedo and Martinez, 2011, 2012; Mar et al., 2011; Luz et al., 2013; Zhang et al., 2014). In addition, PPVs were higher than those reported in literature for the databases included in this study and for fully automatic algorithms. In order to reduce false positives, the exploration of the the integration of an unsupervised learning classifier to the one presented in this work as Llamedo and Martinez (2012) did, could be considered for future work.

## 5. Conclusion

In this work a PAC detector and a classifier for *N*, *S*, and *V* beats is presented. In contrast with many methodologies present in literature, the developed methodology does not require ECG delineation. Although comparison among methodologies presented in different studies is not trivial, the former method outperforms in terms of sensitivity and PPV the state-of-the-art models for PAC detection. Further efforts should be made in order to decrease the inter-patient variability, increase the PPV and reduce false positives so as to be able to use the former method in clinical trials.

## Data Availability Statement

Publicly available datasets were analyzed in this study. Data can be found here: https://physionet.org/about/database/.

## Author Contributions

GG-I conducted the experiments, obtained the results, and contributed most to the writing of the manuscript. LM and VC contributed to the choice of methods, the design of the experimental protocol, and the polishing of the manuscript. All authors contributed to the article and approved the submitted version.

## Conflict of Interest

The authors declare that the research was conducted in the absence of any commercial or financial relationships that could be construed as a potential conflict of interest.
